# Longitudinal analysis of cigar use patterns among US youth and adults, 2013–2019

**DOI:** 10.1186/s12889-023-16253-y

**Published:** 2023-08-18

**Authors:** Jessica King Jensen, Gregory J. Stoddard, Cristine D. Delnevo, Julie W. Merten, Sunday Azagba

**Affiliations:** 1 Rutgers Institute for Nicotine & Tobacco Studies, Rutgers Biomedical and Health Sciences, 303 George Street, New Brunswick, NJ 08901 USA; 2 Family Medicine & Community Health, Rutgers Robert Wood Johnson Medical School, Rutgers Biomedical and Health Sciences, 303 George St, New Brunswick, NJ 08901 USA; 3 Department of Internal Medicine, University of Utah, 30 N 1900 E, Salt Lake City, UT 84132 USA; 4 Brooks College of Health, University of North Florida, 1 UNF Dr Building 39, Jacksonville, FL 32224 USA; 5 Ross and Carol Nese College of Nursing, Penn State University, Nursing Sciences Building, University Park, PA 16802 USA; 6 Social Science Research Institute, Penn State University, 114 Building, Henderson Drive, University Park, PA 16802 USA

**Keywords:** Substance Use and Addiction, Public Health, Tobacco Control

## Abstract

**Background:**

Cigars are available in a range of pack quantities, which contrasts regulations requiring cigarettes to be sold in packs of 20 or greater. Smaller packages may be associated with increases in initiation while larger packs may lead consumers to smoke more. The purpose of this study was to inform pack quantity regulations by examining whether usual cigar pack quantity purchased was associated with use, initiation, and discontinuation among youth and adults for four cigar types: premium cigars, large cigars, cigarillos, and filtered cigars.

**Methods:**

We analyzed waves 1–5 (2013–2019) of the adult and waves 2–5 (2014–2019) of the youth Population Assessment of Tobacco and Health (PATH) Study. Samples included those responding to the item on pack quantity and providing data at all waves (adults: premium cigars [*N* = 536], large cigars [*N* = 1,272], cigarillos [*N* = 3,504], filtered cigars [*N* = 1,281]; youth: premium cigars [*N* = 55], large cigars [*N* = 217], cigarillos [*N* = 1514], filtered cigars [*N* = 266]). Generalized estimating equation models examined the population-averaged effects of pack quantity on cigar use, initiation, and discontinuation.

**Results:**

Adult pack quantity was positively associated with the days used per month for premium cigars (*b*: 0.23, 95% CI: 0.11, 0.34), large cigars (*b*: 0.17, 95% CI: 0.08, 0.25), cigarillos (*b*: 0.12, 95% CI: 0.003, 0.24), and filtered cigars (*b*: 0.07, 95% CI: 0.04, 0.10), and positively associated with amount smoked per day for all cigar types. Youth pack quantity was positively associated with days used per month for premium cigars (*b*: 0.88, 95% CI: 0.33, 1.43), large cigars (*b*: 0.79, 95% CI: 0.43, 1.15), and cigarillos (*b*: 0.17, 95% CI: 0.01, 0.34). Adult initiation was associated with pack quantity for filtered cigars (*b*: -2.22, 95% CI: -4.29, -0.13), as those who initiated purchased smaller pack quantities compared to those who did not initiate that wave. Pack quantity was not associated with discontinuation for adults or youth.

**Conclusions:**

Cigar use increased as usual pack quantity purchased increased across cigar types for youth and adults. Small increases in pack quantity (e.g., one additional cigar) are likely to result in consuming less than one additional day per month, though larger increases (e.g., 10 additional cigars per pack) may result in greater use.

**Supplementary Information:**

The online version contains supplementary material available at 10.1186/s12889-023-16253-y.

## Background

Nearly 1 million youth and 11 million adults in the US reported past 30-day cigar use in 2020, with rates highest among youth and young adults, Black populations, and low socioeconomic status populations [1–3]. Cigar smoking is associated with increased all-cause mortality and increased risk of tobacco-related cancers, heart disease, and stroke, resulting in a significant health disparity for the populations that use cigars [4–7]. Cigars are a product class, with subtypes varying based on product characteristics, user characteristics, price, and pack quantity. While definitions of cigar types can blur distinctions, premium and large cigars are generally more expensive. Adults who use premium and large cigars are typically older, White, and less than 10% report daily use [8, 9]. Cigarillos are mid-sized, often used for cannabis consumption (also commonly described as blunt use), and half of adults purchase singles [10, 11]. Adults who use cigarillos are more likely to be young adults, Black, and approximately 1 in 5 report daily use [8, 9]. Filtered cigars are similar to cigarettes in size, shape, and use, with more adults purchasing 20-packs [8–10]. Adults who use filtered cigars are more likely to be older and smoke more per day, with over one-third reporting daily use [8–10].

Smaller packaging is perceived as easier to conceal and less expensive, making it particularly appealing to youth and low income populations [12]. To reduce youth cigarette use, the United States Food and Drug Administration (US FDA) mandated a minimum pack quantity of 20 for all cigarette products [13]. Some US municipalities have similarly enacted minimum pack quantity policies to reduce the availability of inexpensive cigars [14]. However, cigarette pack quantity is positively associated with consumption, with some consumers preferring to pay a premium for smaller packs to self-regulate their use [15, 16]. Recognizing this relationship, some countries have enacted maximum pack quantity regulations to reduce consumption and limit the affordability of each individual stick [17]. When examining the role of pack quantity as a tobacco control strategy, there is a need to identify the threshold for reducing initiation and use without increasing use among people who continue to purchase the product.

There is limited extant literature on the relationship between cigar pack quantity and cigar use, with studies showing that an increase in cigar pack quantity was associated with an increase in cigars smoked per month for filtered cigars, [10, 18] cigarillos, [18] and traditional cigars [18]. Additionally, adult past-year new use of filtered cigars was associated with purchasing smaller pack quantities [10]. We extend these studies in three important ways: (1) examining the relationship among youth, (2) differentiating between large cigars and premium cigars, and (3) using more longitudinal data points to capture cigar use patterns such as initiation.

## Methods

### Data source

Data were from the Population Assessment of Tobacco and Health (PATH) Study, a nationally representative, longitudinal cohort study [19]. To recruit a nationally representative sample, the PATH study used a four-stage stratified area probability design in Wave 1, conducted September 12, 2013, through December 14, 2014. A total of 32,320 adults, 13,651 youth, and 13,588 parents of youth were interviewed. All Wave 1 respondents still living in the US and not incarcerated were eligible for later annual waves. IRB approval and informed consent were obtained by Westat. Wave 5 weights were used in the present analyses using restricted-use files.

### Study samples

Adults 18 and older at Wave 1 who reported cigar pack quantity purchased at any wave and responded to all five waves were included in the adult sample for all waves. There were slight variations in who received the pack quantity purchase item by wave, with only those who purchased in person receiving it in Wave 1, compared to all purchase locations in subsequent waves. There were additional variations in who received the pack quantity item based on frequency of cigar use (Supplemental Table 1). The adult analyses included 536 adults for premium cigars, 1,272 adults for large cigars, 3,504 adults for cigarillos, and 1,281 adults for filtered cigars. The youth sample included participants under 18 at Wave 1 who reported pack quantity at Waves 2–5 and responded to all waves. Because cigar pack quantity was not asked of youth in Wave 1, youth analyses began in Wave 2. Cigar pack quantity was asked of youth who reported “not light” use in the past 30 days. Youth analyses included 55 youth for premium cigars, 217 youth for large cigars, 1,514 youth for cigarillos, and 266 youth for filtered cigars.

### Measures

#### Cigar type

The PATH Study contains separate items for traditional cigars, cigarillos, and filtered cigars, including separate items differentiating between those who use cigars for exclusive cannabis use and those who do not. We combined exclusive cannabis use and non-exclusive use cigar items for each cigar type as prior analyses identified negligible differences, [18] and regulations would likely apply regardless of reason for use. We separated traditional cigars into premium and large cigars based on brand, [20] or for those not reporting brand, purchase price above $2.00 per cigar [9]. This resulted in four cigar types: premium cigars, large (non-premium) cigars, cigarillos, and filtered cigars. Specific variables used for all measures are noted in Supplemental Table 2, with specific wording and exposure available in the PATH Study Codebooks [19].

#### Pack quantity

For each cigar type, participants were asked to report whether they usually purchase cigars by the box or pack, or as a single cigar. Those who usually purchased cigars by the box or pack were asked to report the number of cigars that came in the box or pack they usually bought. These items were combined to generate a continuous pack quantity variable. We took a conservative approach to outliers, winsorizing pack quantities above 200 (a standard carton of filtered cigars), which resulted in 2 adult cases for large cigars, 2 adult cases and 1 youth case for cigarillos, and 2 adult cases for filtered cigars.

#### Cigar use

For the adult analyses, we had two measures of use, including the number of days of use in the past 30 days (Days Used) and the number of cigars used on each day smoked (Amount Used). For the amount used per day, we winsorized responses greater than 30 for large cigars (*n* = 16), and greater than 40 for cigarillos (*n* = 11) and filtered cigars (*n* = 11). For youth analyses, we had one measure of use, the number of days of use in the past 30 days (Days Used).

#### Discontinuation

For adult and youth analyses, discontinuing use was defined as reporting past 30-day use in the present wave and reporting no past 30-day use in the subsequent wave [21]. Past 30-day use was created based on a combination of items asked and derived (Supplemental Table 2). Discontinuation was analyzed as a categorical outcome (no/yes).

#### Initiation

For adult and youth analyses, initiation was defined based on the derived variable for each cigar type noting respondents who went from never use to ever use at the current wave. Initiation was treated as a predictor (no/yes), with pack quantity a continuous outcome.

#### Sociodemographic characteristics

Adjusted models account for age (continuous), sex (male or female), race (White, Black, other), ethnicity (not Hispanic, Hispanic), income, and residing in a state with a local cigar pack policy as these factors have been associated with pack quantity purchase and use [16, 22].

#### Tobacco use characteristics

Adjusted models account for brand, other tobacco product use, flavor use, and price paid as these factors have been associated with pack quantity purchase and use [16, 22]. Models also adjusted for residing in a state with local cigar minimum pack quantity or pricing regulations, which may influence cigar availability and price [14]. Price was winsorized for adults at above $500 for premium cigars (*n* = 2) and large cigars (*n* = 5), above $108.50 for cigarillos (*n* = 17), and above $500.23 for filtered cigars (*n* = 5). For youth, price was winsorized for values above $200 (*n* = 4 cigarillo cases).

### Analyses

Complex survey weights were applied to all models to accommodate the multi-stage probability design. Sociodemographic characteristics were assessed for each cigar type for both adults and youth. Weighted counts and percentages were assessed for categorical variables, and weighted means and standard deviations were calculated for continuous variables.

For each cigar type, a generalized estimating equation (GEE) model, with an independent correlation structure, was used to examine the population-averaged (marginal) effects of the independent variable (i.e., pack quantity or initiation) on the dependent variable (i.e., days used, amount used, discontinuation), while accounting for repeated measurements across years (waves). The five waves of data provided five repeated measurements per survey respondent (four for youth analyses). Our models were not specified to test for linear trends over time or make pairwise comparisons between waves. Instead, the models were five measurements clustered, or nested, within each respondent. The measurements represent time-varying covariates, with possibly different values at each wave. The association of variables within each wave were modeled and the reported regression coefficients are the average associations across the waves. Multicollinearity between predictor variables was assessed when constructing our multivariable models, leaving one of two highly correlated variables out of the model if multicollinearity presented an issue. Models adjusted for sociodemographic and tobacco use characteristics. Complex survey weights were applied using the balanced repeated replication method with Fay’s adjustment of 0.3, as recommended by the PATH Study team [19]. All tests were two-sided, *p*-values < 0.05 were considered significant, and analyses were conducted in 2022–2023 using SPSS 28 and Stata 17.

## Results

### Sample characteristics

Tables 1 and 2 contain the sociodemographic and cigar use characteristics for the samples. For the adult premium cigar sample (*n* = 536 adults, *n* = 1,070 observations), the mean age was 40.4 (SE = 0.8), 95.8% were male, 82.5% were White, the mean usual pack quantity purchased was 5.6 (SE = 0.4), and the mean days used per month was 4.3 (SE = 0.7). Of 433 respondents with pack quantity at multiple waves, most (62.4%) reported the same pack quantity, 13.4% decreased, 8.7% increased, and 15.6% increased and decreased their reported pack quantities. For adults who used large cigars (*n* = 1,272 adults; *n* = 2,241 observations), the mean age was 36.2 (SE = 0.6), 78.4% were male, 64.7% were White, the mean pack quantity was 4.9 (SE = 0.4), and the mean days used per month was 8.6 (SE = 0.3). Of 786 respondents with pack quantity at multiple waves, 51.2% reported the same pack quantity, 16.2% decreased, 17.0% increased, and 15.7% increased and decreased their reported pack quantities. For adults who used cigarillos (*n* = 3,504 adults; *n* = 9,552 observations), the mean age was 31.9 (SE = 0.3), 66.0% were male, 62.7% were White, the mean pack quantity was 3.0 (SE = 0.1), and the mean days used per month was 8.7 (SE = 0.2). Of 2,569 respondents with pack quantity at multiple waves, 42.9% reported the same pack quantity, 17.3% decreased, 20.9% increased, and 18.9% increased and decreased their reported pack quantities. For adults who used filtered cigars (*n* = 1,281 adults; *n* = 2,485 observations), the mean age was 37.8 (SE = 0.5), 66.7% were male, 65.5% were White, the mean pack quantity was 13.0 (SE = 0.7), and the mean days used per month was 11.2 (SE = 0.5). Of 530 respondents with pack quantity at multiple waves, 50.2% reported the same pack quantity, 22.0% decreased, 13.5% increased, and 14.3% increased and decreased their reported pack quantities.

**Table 1:** Adult and youth sample sociodemographic characteristics at initial wave of analysis

**Adult Characteristics at Wave 1 (2013–2014)**	**Premium Cigar** ***N*** **= 536**	**Large Cigar** ***N*** **= 1,272**	**Cigarillo** ***N*** **= 3,504**	**Filtered Cigar** ***N*** **= 1,281**
Age (Mean [SE])	40.4 (0.8)	36.2 (0.6)	31.9 (0.3)	37.8 (0.5)
Sex (n [%])				
Female	32 (4.2)	357 (21.6)	1432 (34.0)	515 (33.3)
Male	504 (95.8)	915 (78.4)	2072 (66.0)	766 (66.7)
Race (n [%])				
White alone	426 (82.5)	743 (64.7)	1966 (62.7)	758 (65.5)
Black alone	52 (9.1)	376 (26.7)	1059 (28.3)	338 (24.6)
Asian alone	7 (2.9)	12 (2.1)	30 (1.8)	16 (2.6)
Other	45 (5.4)	117 (6.5)	365 (7.2)	135 (7.3)
Ethnicity (n [%])				
Not Hispanic	474 (89.7)	1070 (87.2)	2791 (82.3)	1005 (81.9)
Hispanic	62 (10.3)	186 (12.8)	678 (17.7)	254 (18.1)
Income				
< $25,000	120 (16.9)	651 (42.4)	1946 (42.7)	778 (53.8)
$25,000-$49,999	94 (15.6)	243 (19.6)	674 (17.3)	233 (17.9)
$50,000-$99,999	140 (25.1)	182 (13.6)	401 (10.5)	116 (8.5)
$100,000 +	150 (28.1)	107 (11.0)	226 (6.8)	48 (3.8)
Not provided	84 (14.3)	271 (13.4)	1635 (22.8)	349 (16.1)
**Youth Characteristics at Wave 2 (2014–2015)**	**Premium Cigar** ***N* = 55**	**Large Cigar** ***N* = 217**	**Cigarillo** ***N* = 1,514**	**Filtered Cigar** ***N* = 266**
Age (Mean [SE])	16.6 (0.1)	16.5 (0.1)	16.4 (0.04)	16.4 (0.1)
Sex (n [%])				
Female	4 (4.6)	51 (22.3)	663 (42.4)	95 (33.6)
Male	51 (95.4)	166 (77.7)	851 (57.6)	171 (66.4)
Race (n [%])				
White alone	47 (90.4)	133 (68.7)	904 (68.5)	162 (70.2)
Other	8 (9.6)	74 (31.3)	539 (31.4)	89 (29.8)
Ethnicity (n [%])				
Not Hispanic	47 (91.4)	167 (82.2)	1120 (80.0)	200 (83.1)
Hispanic	8 (8.6)	48 (17.8)	385 (20.0)	60 (16.9)

Notes: Samples include respondents who reported pack quantity and had data at each wave

*SE* Standard error

**Table 2:** Adult and youth cigar use characteristics by product

**Adult Cigar Use Behaviors at Waves 1–5 (2013–2019)**	**Premium Cigar** ***N*** **= 1,070**	**Large Cigar** ***N*** **= 2,241**	**Cigarillo** ***N*** **= 9,552**	**Filtered Cigar** ***N*** **= 2,485**
Pack quantity (Mean [SE])	5.6 (0.4)	4.9 (0.4)	3.0 (0.1)	13.0 (0.7)
Pack quantity (n [%])				
Single	780 (71.0)	1306 (58.7)	5195 (54.2)	881 (32.2)
2–5-pack	55 (5.2)	664 (27.3)	3785 (38.8)	342 (12.0)
6–19-pack	65 (5.9)	105 (5.0)	287 (3.6)	266 (11.3)
20+ pack	171 (17.9)	166 (9.0)	291 (3.4)	996 (44.5)
Cigar Use (Mean [SE])				
Days used	4.3 (0.7)	8.6 (0.3)	8.7 (0.2)	11.2 (0.5)
Amount used	0.7 (0.1)	1.6 (0.1)	1.5 (0.05)	4.0 (0.2)
Discontinuation (n [%])				
Continued use	857 (80.9)	1561 (70.0)	7457 (76.9)	1855 (73.5)
Discontinued use	213 (19.1)	680 (30.0)	2095 (23.1)	630 (26.5)
Initiation (n [%])				
Continued use	1027 (96.7)	1907 (87.0)	8769 (93.3)	1983 (80.5)
Initiated use	43 (3.3)	334 (13.0)	783 (6.7)	502 (19.5)
Other Tobacco Use (n [%])				
No OTP use	452 (47.9)	290 (16.8)	2287 (23.0)	211 (8.3)
OTP use	618 (52.1)	1947 (83.2)	7265 (77.0)	2274 (91.7)
Flavors (n [%])				
No/Don’t know	–	1535 (71.1)	5947 (63.9)	1545 (61.8)
Use flavors	--	706 (28.9)	3605 (36.1)	940 (38.2)
Price Per Pack (Mean [SE])	$31.60 (3.82)	$8.88 (0.92)	$2.87 (0.12)	$6.62 (0.82)
Price Per Cigar (Mean [SE])	$8.41 (0.30)	$3.00 (0.19)	$1.19 (0.02)	$1.19 (0.09)
Local Policy in State (n [%])				
No policies in state	882 (82.9)	1919 (85.3)	8007 (83.2)	2200 (87.9)
1 or more policies	188 (17.1)	322 (14.7)	1545 (16.8)	285 (12.1)
**Youth Cigar Use Behaviors at Waves 2–5 (2014–2019)**	**Premium Cigar** ***N* = 67**	**Large Cigar** ***N* = 253**	**Cigarillo** ***N* = 2,413**	**Filtered Cigar** ***N* = 318**
Pack quantity (Mean [SE])	1.8 (0.3)	2.7 (0.3)	2.1 (0.1)	6.6 (0.7)
Pack quantity (n [%])				
Single	59 (89.7)	148 (57.5)	1340 (54.6)	164 (48.9)
2–5-pack^a^	8 (10.3)^a^	88 (36.0)	994 (42.3)	69 (21.2)
6–19-pack	--	10 (3.6)	37 (1.6)	25 (8.8)
20+ -pack	--	7 (2.9)	42 (1.5)	60 (21.0)
Cigar Use (Mean [SE])				
Days used	3.3 (0.7)	8.2 (0.8)	7.1 (0.3)	5.5 (0.6)
Discontinuation (n [%])				
Continued use	47 (71.0)	169 (65.1)	1884 (77.8)	229 (69.9)
Discontinued use	20 (29.0)	84 (34.9)	529 (22.2)	89 (30.1)
Initiation (n [%])				
Continued use	43 (63.1)	172 (67.2)	1914 (80.0)	187 (58.0)
Initiated use	24 (36.9)	81 (32.8)	499 (20.0)	131 (42.0)
Other Tobacco Use (n [%])				
No OTP use	21 (33.2)	29 (11.6)	854 (33.8)	32 (9.0)
OTP use	46 (66.7)	224 (88.4)	1559 (66.2)	284 (91.0)
Flavors (n [%])				
No/Don’t know	–	164 (65.6)	1376 (57.6)	235 (74.4)
Use flavors	–	89 (34.4)	1037 (42.4)	83 (25.6)
Price Per Pack (Mean [SE])	$10.99 (1.42)	$5.08 (0.79)	$2.40 (0.21)	$3.85 (0.51)
Price Per Cigar (Mean [SE])	$8.19 (0.58)	$2.58 (0.31)	$1.44 (0.15)	$1.81 (0.39)
Local Policy in State (n [%])				
No policies in state	53 (77.9)	210 (81.3)	2004 (82.9)	280 (87.8)
1 or more policies	14 (22.1)	43 (18.7)	409 (17.1)	38 (12.2)

Notes: Samples include respondents who reported pack quantity and had data at each wave

*SE* Standard error

^a^ Due to small cell counts for premium cigars by pack quantities, 2+ packs were combined

For the youth premium cigar sample (*n* = 55 youth; *n* = 67 observations), the mean age was 16.6 (SE = 0.1), 95.4% were male, 90.4% were White, the mean usual pack quantity purchased was 1.8 (SE = 0.3), and the mean days used per month was 3.3 (SE = 0.7). For the youth large cigar sample (*n* = 217 youth; *n* = 253 observations), the mean age was 16.5 (SE = 0.1), 77.7% were male, 68.7% were White, the mean pack quantity was 2.7 (SE = 0.3), and the mean days used per month was 8.2 (SE = 0.8). In the youth cigarillo sample (*n* = 1,514 youth; *n* = 2,413 observations), the mean age was 16.4 (SE = 0.04), 57.6% were male, 68.5% were White, the mean pack quantity was 2.1 (SE = 0.1), and the mean days used per month was 7.1 (SE = 0.3). In the youth filtered cigar sample (*n* = 266 youth; *n* = 318 observations), the mean age was 16.4 (SE = 0.1), 66.4% were male, 70.2% were White, the mean pack quantity was 6.6 (SE = 0.7), and the mean days used per month was 5.5 (SE = 0.6).

### Pack quantity and use

Adult usual pack quantity was positively associated with days used for premium cigars (per 1 additional cigar, *b*: 0.23, 95% CI: 0.11, 0.34), large cigars (*b*: 0.17, 95% CI: 0.08, 0.25), cigarillos (*b*: 0.12, 95% CI: 0.003, 0.24), and filtered cigars (*b*: 0.07, 95% CI: 0.04, 0.10; Table 3). In other words, for each 10 additional cigars in the usual purchase pack, adults smoked an additional 2.3 days for premium cigars, 1.7 days for large cigars, 1.2 days for cigarillos, and 0.7 days for filtered cigars. Usual pack quantity purchased was positively associated with the amount used per day for premium cigars (per 1 additional cigar, *b*: 0.02, 95% CI: 0.001, 0.03; Table 3), large cigars (*b*: 0.04, 95% CI: 0.01, 0.07), cigarillos (*b*: 0.08, 95% CI: 0.02, 0.13) and filtered cigars (*b*: 0.05, 95% CI: 0.02, 0.08; Table 3).

**Table 3:** Associations between usual pack quantity purchased and cigar use behaviors among adults, waves 1–5 (2013–2019)

	**Days Used Per Month** Adjusted *b* (95% CI)	**Amount Used Per Day** Adjusted *b* (95% CI)	**Discontinuation** Adjusted OR (95% CI)	**Initiation** Adjusted *b* (95% CI)
Premium Cigars	**0.23 (0.11, 0.34)**	**0.02 (0.001, 0.03)**	0.97 (0.93, 1.01)	-0.92 (-2.39, 0.54)
Large Cigars	**0.17 (0.08, 0.25)**	**0.04 (0.01, 0.07)**	0.99 (0.97, 1.001)	-0.14 (-1.18, 0.91)
Cigarillos	**0.12 (0.003, 0.24)**	**0.08 (0.02, 0.13)**	0.99 (0.97, 1.01)	0.28 (-0.16, 0.73)
Filtered Cigars	**0.07 (0.04, 0.10)**	**0.05 (0.02, 0.08)**	1.00 (0.99, 1.004)	**-2.21 (-4.29, -0.13)**

Notes: For Use and Discontinuation variables, pack quantity was the predictor; for Initiation, pack quantity was the outcome. Models adjust for wave, age, sex, race, ethnicity, other tobacco product use, brand, price paid, flavor use (not premium cigars), and cigar pack or price policy within the state. Adjusted *b* is the adjusted mean increase in the outcome (column heading) per 1 cigar increase in pack quantity

Youth pack quantity was positively associated with days used for premium cigars (per 1 additional cigar, *b*: 0.88, 95% CI: 0.33, 1.43), large cigars (*b*: 0.79, 95% CI: 0.43, 1.15), and cigarillos (*b*: 0.17, 95% CI: 0.17, 0.01, 0.34; Table 4). For each 10 additional cigars in the usual purchase pack, youth smoked an additional 8.8 days for premium cigars, 7.9 days for large cigars, and 1.7 days for cigarillos per month. Filtered cigar pack quantity was not associated with days used.

**Table 4:** Associations between usual pack quantity purchased and cigar use behaviors among youth, waves 2–5 (2014–2019)

	**Days Used Per Month** Adjusted *b* (95% CI)	**Discontinuation** Adjusted OR (95% CI)	**Initiation** Adjusted *b *(95% CI)
Premium Cigars	**0.88 (0.33, 1.43)**	0.97 (0.88, 1.06)	-0.13 (-1.47, 1.20)
Large Cigars	**0.79 (0.43, 1.15)**	1.11 (0.92, 1.33)	0.32 (-0.75, 1.40)
Cigarillos	**0.17 (0.01, 0.34)**	1.01 (0.97, 1.05)	0.24 (-0.19, 0.67)
Filtered Cigars	0.10 (-0.09, 0.29)	0.95 (0.86, 1.05)	0.28 (-1.98, 2.54)

Notes: For days used per month and discontinuation, pack quantity was the predictor; for Initiation, pack quantity was the outcome. Models adjust for wave, age, sex, race, ethnicity, other tobacco product use, brand, price paid, flavor use (not premium cigars), and cigar pack or price policy within the state. Adjusted *b* is the adjusted mean increase in the outcome (column heading) per 1 cigar increase in pack quantity

### Pack quantity and discontinuation

In adjusted models, usual pack quantity was not associated with discontinuing use for any cigar types for adults or youth (Tables 3 and 4).

### Initiation and pack quantity

Adult initiation was associated with pack quantity for filtered cigars (*b*: -2.21, 95% CI: -4.29, -0.13; Table 3). Adults who initiated filtered cigar use purchased smaller pack quantities compared to those who did not initiate that wave. In adjusted models, initiation was not associated with pack quantity for any other cigar types for adults or youth (Tables 3 and 4).

## Discussion

In this nationally representative study using longitudinal US data, we identified associations between usual cigar pack quantity purchased and cigar use across cigar types for youth and adults. For youth, pack quantity purchased was positively associated with the number of days used per month for premium cigars, large cigars, and cigarillos. For adults, pack quantity purchased was positively associated with the number of days used per month the number of cigars smoked per day for all cigar types. Few associations were identified for discontinuation and initiation.

Our findings extend prior work in critical ways. First, our analyses differentiate between premium and large cigars. Compared to prior analyses which excluded or combined premium cigars with large cigars, we examined all outcomes for both youth and adults. With premium cigars previously under consideration for exclusion from FDA regulation [23] and the recent National Academies of Science, Engineering, and Medicine report emphasizing the need for research on premium cigars, [20] it is important to obtain specific data on this cigar type. Our findings show that for premium cigars, like other cigar types, purchasing larger pack quantities is associated with consuming more. Second, this study presents the first data on examining pack quantity purchase and use among youth. Youth represent a vulnerable population as defined by the NIH, and having youth-specific data to inform regulation is critical. Findings provide evidence that youth cigar pack quantity purchase is associated with use, such that as pack quantity purchased increases, so does the number of days used per month.

While there is a consistent positive association in pack quantity and use, the increases appear to be minimal to modest. Each additional cigar within a package would result in less than one additional day of use per month for youth. For adults, we identified an increase in smoking of less than a quarter of a day per month for each additional cigar in the usual purchase package for any of the cigar types. This may indicate that switching from purchasing a 4-pack of cigars to a 5-pack of cigars may have minimal impact on health outcomes. That said, switching from purchasing singles to 10-packs or 20-packs, as some local US cigar pack quantity policies have set as the minimum pack quantity for sale, [14] could result in larger increases in smoking.

Our findings align with our prior analyses of Waves 1–3 and with tobacco industry documents that indicate purchasing larger pack quantities is associated with smoking more days per month and smoking more cigars per day [12, 18]. In contrast, purchasing smaller pack quantities is associated with reduced use, which may have implications for improving public health. While the greatest benefits to health are seen with cessation or substantial reductions, reducing smoking has been associated with improvements in respiratory symptoms [24, 25].

Initiation was only associated with filtered cigar pack quantity purchase among adults. Based on industry documentation and other studies showing a relationship between smoking history and use, [12, 22] we would expect youth and adults who initiate with a product would report purchasing smaller pack quantities. However, it is worth noting that question branching in the PATH Study make assessing the role of cigar pack quantity on initiation challenging. First, the pack quantity item assessed usual purchase quantity, not the quantity initiated with. It is possible youth and adults initiated with a smaller pack quantity and transitioned to purchasing larger pack quantities by the time the survey was conducted. Second, the pack quantity item is only asked of youth who reported “not light” past 30-day use, which may not capture those experimenting with use. It is also possible that youth and adults categorized as initiating use for each specific cigar type had used cigarettes or other types of cigars, given high rates of other tobacco use among the samples.

Concerning discontinuing use, we did not find any significant associations for youth or adults after adjusting for sociodemographic and use characteristics. This aligns with Persoskie and colleagues’ analysis of Waves 1 and 2, which identified a significant association in unadjusted analyses for large cigars but not adjusted analyses [10]. Experiments with adults who smoke cigarettes found they preferred smaller packaging to self-regulate their smoking behaviors or quit smoking [15]. Similarly, adults report preferring smaller cigar packaging to avoid consuming more [16]. However, recent studies have shown that a sharp reduction in use is more effective than a gradual approach by reducing smoking behavior over time [26, 27]. Adults who want to quit smoking may stop purchasing cigars altogether instead of changing their purchasing behaviors.

### Limitations

There are several limitations to note. First, data were self-reported and may be subject to bias or error based on social desirability, recall bias, pack quantity misclassification, or cigar-type misclassification. However, we compared self-reported pack quantity responses to Nielsen retailer sales data on pack quantity to confirm response plausibility. Additionally, the PATH Study team attempts to obtain the actual product from participants and provides images of various cigar types to reduce misclassification. Second, based on the available measures and analyses conducted, we cannot determine a causal relationship between cigar pack quantity purchased and use; the identified associations may reflect that amount smoked influences purchase quantity. Third, cigar pack quantity is only asked of those who report current or non-light use, limiting insights among those who smoke less frequently and our ability to assess patterns of use among those experimenting with cigar use. Fourth, polytobacco use across cigar types and with cigarette use is common. While other tobacco use was adjusted for in the present study, examining patterns by exclusive cigar type use and initiation was not feasible. A prior study examining substitution identified no significant associations between cigar pack quantity and cigarette or cigar substitution in adjusted models, [10] though this should be considered in future studies.

## Conclusions

The current study found that premium cigar, large cigar, cigarillo, and filtered cigar pack purchase quantity were positively associated with adult cigar use, and premium cigar, large cigar, and cigarillo pack quantity purchased were positively associated with youth cigar use. As pack quantity increases, so does use, though the magnitude of the impact may be minimal for each additional cigar. Given the authority of the US FDA and state and local jurisdictions over cigar pack quantity, this study provides data pertinent to potential minimum and maximum package quantity regulations which may need to include a combination of pack quantity and pricing restrictions to best regulate use.

### Supplementary Information

**Additional file 1: eTable 1.** Adults and youth who received the pack quantity item at each wave.** eTable 2.** PATH study variables used in analyses.

## Data Availability

Data are available following approval and completion of a Restricted Data Use Agreement with the Inter-university Consortium for Political and Social Research. Variables used in the analyses are in Supplemental Table 2. Analytic code available upon reasonable request to corresponding author.

## Abbreviations

US: United States
FDA: Food and Drug Administration
PATH: Population Assessment of Tobacco and Health
GEE: Generalized estimating equation
