# Supplementary material for: Longitudinal analysis of cigar use patterns among US youth and adults, 2013–2019
Source: BMC Public Health. 2023 Aug 18;23:1580. doi: 10.1186/s12889-023-16253-y (PMC10439534; doi:10.1186/s12889-023-16253-y)
Supplement: Supplementary file 1 — Additional file 1: eTable 1. Adults and youth who received the pack quantity item at each wave. eTable 2. PATH study variables used in analyses. [file 12889_2023_16253_MOESM1_ESM.docx]

| **eTable 1. Adults and Youth who Received the Pack Quantity Item at Each Wave** | | | | | | |
| --- | --- | --- | --- | --- | --- | --- |
|  | Wave 1 | Wave 2 | Wave 3 | Wave 4 | Wave 5 |  |
| Adult Pack Quantity for non-exclusive blunt use | Current [cigar type] smokers or blunt-only [cigar type] smokers who usually buy [cigar type] in person. | Adult respondents who are current established [cigar type] smokers or blunt-only [cigar type] smokers and usually buy [cigar type] in person, from the internet, or by telephone. | Adult respondents who are current established, current experimental, or non-current 30-day [cigar type] smokers and usually buy their [cigar type] in person, from the internet, or by telephone. | Adult respondents who are current established, current experimental, or non-current 30-day [cigar type] smokers and are not past 12 month blunt-only [cigar type] smokers and usually buy their [cigar type] in person, from the internet, or by telephone. | Adult respondents who are current established, current experimental, or non-current 30-day [cigar type] smokers and are not past 12 month blunt-only [cigar type] smokers and usually buy their [cigar type] in person, from the internet, or by telephone. |  |
| Adult Pack Quantity for exclusive blunt use | Same as above | Same as above | Adult respondents who are past 12 month blunt-only [cigar type] smokers and usually buy their [cigar type] for blunts in person, from the internet, or by telephone. | Adult respondents who are past 12 month blunt-only [cigar type] smokers and usually buy their [cigar type] for blunts in person, from the internet, or by telephone. | Adult respondents who are past 12 month blunt-only [cigar type] smokers and usually buy their [cigar type] for blunts in person, from the internet, or by telephone. |  |
| Youth Pack Quantity for non-exclusive blunt use | Not assessed | Youth respondents who are past 30 day "not-light" [cigar type] smokers and usually get [cigar type] by giving someone else money to buy for them or buying themselves from a store, or youth respondents who are past 30 day blunt-only [cigar type] smokers. | Youth respondents who are past 30 day "not-light" [cigar type] smokers and usually get [cigar type] by giving someone else money to buy for them or buying themselves from a store. | Youth respondents who are past 30 day "not-light" [cigar type] smokers and usually get [cigar type] by giving someone else money to buy for them or buying themselves from a store. | Youth respondents who are past 30 day "not-light" [cigar type] smokers and usually get [cigar type] by giving someone else money to buy for them or buying themselves from a store. |  |
| Youth Pack Quantity for exclusive blunt use | Not assessed | Same as above | Youth respondents who are past 30 day blunt-only [cigar type] smokers and usually get [cigar type] for blunts by giving someone else money to buy for them or buying themselves from a store. | Youth respondents who are past 30 day blunt-only [cigar type] smokers and usually get [cigar type] for blunts by giving someone else money to buy for them or buying themselves from a store. | Youth respondents who are past 30 day blunt-only [cigar type] smokers and usually get [cigar type] for blunts by giving someone else money to buy for them or buying themselves from a store. |  |

| **eTable 2. PATH Study Variables Used in Analyses** | | | |
| --- | --- | --- | --- |
| **Construct** | **Age** | **PATH Variables Used** | **Recoding Notes** |
| Pack Size | Adult | R01_AG1039TC, R01_AG1039CG, R01_AG1039FC, R02_AG1039TC, R02_AG1039TJ, R02_AG1039CG, R02_AG1039GJ, R02_AG1039FC, R02_AG1039FJ, R03_AG1039TC, R03_AJ1039TC, R03_AG1039CG, R03_AJ1039CG, R03_AG1039FC, R03_AJ1039FC, R04_AG1039TC, R04_AJ1039TC, R04_AG1039CG, R04_AJ1039CG, R04_AG1039FC, R04_AJ1039FC, R05_AG1039TC, R05_AJ1039TC, R05_AG1039CG, R05_AJ1039CG, R05_AG1039FC, R05_AJ1039FC  R01_AG1040TC, R01_AG1040CG, R01_AG1040FC, R02_AG1040TC, R02_AG1040TJ, R02_AG1040CG, R02_AG1040GJ, R02_AG1040FC, R02_AG1040FJ, R03_AG1040TC, R03_AJ1040TC, R03_AG1040CG, R03_AJ1040CG, R03_AG1040FC, R03_AJ1040FC, R04_AG1040TC, R04_AJ1040TC, R04_AG1040CG, R04_AJ1040CG, R04_AG1040FC, R04_AJ1040FC, R05_AG1040TC, R05_AJ1040TC, R05_AG1040CG, R05_AJ1040CG, R05_AG1040FC, R05_AJ1040FC | Combined box or single (1039) and number in box (1040) to create continuous pack size variables for each cigar type.  Combined exclusive blunting variables (J notation) with cigar variables for waves 2-5.  Winsorized 2 cases for each cigar type at 200 |
|  | Youth | R02_YG1039TC, R02_YG1039TJ, R02_YG1039CL, R02_YG1039GJ, R02_YG1039FC, R02_YG1039FJ, R03_YG1039TC, R03_YJ1039TC, R03_YG1039CL, R03_YJ1039CG, R03_YG1039FC, R03_YJ1039FC, R04_YG1039TC, R04_YJ1039TC, R04_YG1039CL, R04_YJ1039CG, R04_YG1039FC, R04_YJ1039FC, R05_YG1039TC, R05_YJ1039TC, R05_YG1039CL, R05_YJ1039CG, R05_YG1039FC, R05_YJ1039FC  R02_YG1040TC, R02_YG1040TJ, R02_YG1040CL, R02_YG1040GJ, R02_YG1040FC, R02_YG1040FJ, R03_YG1040TC, R03_YJ1040TC, R03_YG1040CL, R03_YJ1040CG, R03_YG1040FC, R03_YJ1040FC, R04_YG1040TC, R04_YJ1040TC, R04_YG1040CL, R04_YJ1040CG, R04_YG1040FC, R04_YJ1040FC, R05_YG1040TC, R05_YJ1040TC, R05_YG1040CL, R05_YJ1040CG, R05_YG1040FC, R05_YJ1040FC | Combined aged-up adults to applicable youth variables.  Combined box or single (1039) and number in box (1040) to create continuous pack size variables for each cigar type.  Combined exclusive blunting variables (J notation) with cigar variables.  Winsorized 1 cigarillos case at 200 |
| Days used per month | Adult | R01_AG1022TC, R01_AG1022CG, R01_AG1022FC, R02_AG1022TC, R02_AG1022TJ, R02_AG1022CG, R02_AG1022GJ, R02_AG1022FC, R02_AG1022FJ, R03_AG1022TC, R03_AJ1022TC, R03_AG1022CG, R03_AJ1022CG, R03_AG1022FC, R03_AJ1022FC, R04_AG1022TC, R04_AJ1022TC, R04_AG1022CG, R04_AJ1022CG, R04_AG1022FC, R04_AJ1022FC, R05_AG1022TC, R05_AJ1022TC, R05_AG1022CG, R05_AJ1022CG, R05_AG1022FC, R05_AJ1022FC  R01_AG1003TC, R01_AG1003CG, R01_AG1003FC, R02_AG1003TC, R02_AG1003TJ, R02_AG1003CG, R02_AG1003GJ, R02_AG1003FC, R02_AG1003FJ, R03_AG1003TC, R03_AG1003TJ, R03_AG1003CG, R03_AG1003GJ, R03_AG1003FC, R03_AG1003FJ, R04_AG1003TC, R04_AG1003TJ, R04_AG1003CG, R04_AG1003GJ, R04_AG1003FC, R04_AG1003FJ R05_AG1003TC, R05_AG1003TJ, R05_AG1003CG, R05_AG1003GJ, R05_AG1003FC, R05_AG1003FJ  R01R_A_EDY_GTRAD, R01R_A_EDY_GRILLO, R01R_A_EDY_GFILTR, R02R_A_EDY_GTRAD, R02R_A_EDY_GRILLO, R02R_A_EDY_GFILTR, R03R_A_EDY_GTRAD, R03R_A_EDY_GRILLO, R03R_A_EDY_GFILTR, R04R_A_EDY_GTRAD, R04R_A_EDY_GRILLO, R04R_A_EDY_GFILTR, R05R_A_EDY_GTRAD, R05R_A_EDY_GRILLO, R05R_A_EDY_GFILTR | Combined number of days used (1022) and two variables for everyday use (1003 and EDY) for continuous days used.  Combined exclusive blunting variables (J notation) with cigar variables for waves 2-5. |
|  | Youth | R02_YG1022TC, R02_YG1022TJ, R02_YG1022CL, R03_YG1022TC, R03_YJ1022TC, R03_YG1022CL, R03_YJ1022CG, R03_YG1022FC, R03_YJ1022FC, R04_YG1022TC, R04_YJ1022TC, R04_YG1022CL, R04_YJ1022CG, R04_YG1022FC, R04_YJ1022FC, R05_YG1022TC, R05_YJ1022TC, R05_YG1022CL, R05_YJ1022CG, R05_YG1022FC, R05_YJ1022FC | Combined aged-up adults to applicable youth variables.  Combined exclusive blunting variables (J notation) with cigar variables for waves 3-5. |
| Amount used per day | Adult | R01_AG1023TC, R01_AG1023CG, R01_AG1023FC, R02_AG1023TC, R02_AG1023CG, R02_AG1023FC, R03_AG1023TC, R03_AJ1023TC, R03_AG1023CG, R03_AJ1023CG, R03_AG1023FC, R03_AJ1023FC, R04_AG1023TC, R04_AJ1023TC, R04_AG1023CG, R04_AJ1023CG, R04_AG1023FC, R04_AJ1023FC, R05_AG1023TC, R05_AJ1023TC, R05_AG1023CG, R05_AJ1023CG, R05_AG1023FC, R05_AJ1023FC  R01_AG1021TC, R01_AG1021CG, R01_AG1021FC, R02_AG1021TC, R02_AG1021CG, R02_AG1021FC, R03_AG1021TC, R03_AG1021CG, R03_AG1021FC, R04_AG1021TC, R04_AG1021CG, R04_AG1021FC, R05_AG1021TC, R05_AG1021CG, R05_AG1021FC | Recoded partial use as 0 for those who used some days (1023) and those who smoked every day (1021).  Combined amount used per day for those who used some days (1023) and those who smoked every day (1021).  Combined exclusive blunting variables (J notation) with cigar variables for waves 3-5.  Winsorized: TC: 30 (16 cases); CG: 40 (11 cases); FC: 40 (11 cases) |
| Quit | Adult | R01_AG1004TC, R01_AG1004CG, R01_AG1004FC, R02_AG9030, R02_AG9031, R03_AG9030, R03_AG9031, R04_AG9030, R04_AG9031, R05_AG9030, R05_AG9031 R01R_A_DAYSQUIT_GTRAD, R01R_A_DAYSQUIT_GRILLO, R01R_A_DAYSQUIT_GFILTR, R02_AG1009TC_NN, R02_AG1009TJ_NN, R02_AG1009TC_UN, R02_AG1009TJ_UN, R02_AG1009CG_NN, R02_AG1009GJ_NN, R02_AG1009CG_UN, R02_AG1009GJ_UN, R02_AG1009FC_NN, R02_AG1009FJ_NN, R02_AG1009FC_UN, R02_AG1009FJ_UN, R03R_A_DAYSQUIT_GTRAD, R03R_A_DAYSQUIT_GRILLO, R03R_A_DAYSQUIT_GFILTR, R04R_A_DAYSQUIT_GTRAD, R04R_A_DAYSQUIT_GRILLO, R04R_A_DAYSQUIT_GFILTR, R05R_A_DAYSQUIT_GTRAD, R05R_A_DAYSQUIT_GRILLO, R05R_A_DAYSQUIT_GFILTR  R01R_A_P30D_GTRAD, R01R_A_P30D_GRILLO, R01R_A_P30D_GFILTR, R02R_A_P30D_GTRAD, R02R_A_P30D_GRILLO, R02R_A_P30D_GFILTR, R03R_A_P30D_GTRAD, R03R_A_P30D_GRILLO, R03R_A_P30D_GFILTR, R04R_A_P30D_GTRAD, R04R_A_P30D_GRILLO, R04R_A_P30D_GFILTR, R05R_A_P30D_GTRAD, R05R_A_P30D_GRILLO, R05R_A_P30D_GFILTR  R01_AG1003TC, R01_AG1003CG, R01_AG1003FC, R02_AG1003TC, R02_AG1003CG, R02_AG1003FC, R02_AG1003TJ, R02_AG1003GJ, R02_AG1003FJ, R03_AG1003TC, R03_AG1003CG, R03_AG1003FC, R03_AG1003TJ, R03_AG1003GJ, R03_AG1003FJ, R04_AG1003TC, R04_AG1003CG, R04_AG1003FC, R04_AG1003TJ, R04_AG1003GJ, R04_AG1003FJ, R05_AG1003TC, R05_AG1003CG, R05_AG1003FC, R05_AG1003TJ, R05_AG1003GJ, R05_AG1003FJ  R01_AG1022TC, R01_AG1022CG, R01_AG1022FC, R02_AG1022TC, R02_AG1022CG, R02_AG1022FC, R03_AG1022TC, R03_AG1022CG, R03_AG1022FC, R03_AJ1022TC, R03_AJ1022CG, R03_AJ1022FC, R04_AG1022TC, R04_AG1022CG, R04_AG1022FC, R04_AJ1022TC, R04_AJ1022CG, R04_AJ1022FC, R05_AG1022TC, R05_AG1022CG, R05_AG1022FC, R05_AJ1022TC, R05_AJ1022CG, R05_AJ1022FC | Combined 5 variables for past 30-day use. Quit was defined as reporting past 30 day use in wave N, and no past 30-day use in wave N+1.  Combined exclusive blunting variables (J notation) with cigar variables for waves 2-5. |
|  | Youth | R02_YG1112TC, R02_YG1112TJ, R02_YG1112CL, R02_YG1112GJ, R02_YG1112FC, R02_YG1112FJ, R03_YG1112TC, R03_YJ1112TC, R03_YG1112CL, R03_YJ1112CG, R03_YG1112FC, R03_YJ1112FC, R04_YG1112TC, R04_YJ1112TC, R04_YG1112CL, R04_YJ1112CG, R04_YG1112FC, R04_YJ1112FC, R05_YG1112TC, R05_YJ1112TC, R05_YG1112CL, R05_YJ1112CG, R05_YG1112FC, R05_YJ1112FC  R02_YG1022TC, R02_YG1022CL, R02_YG1022FC, R03_YG1022TC, R03_YJ1022TC, R03_YG1022CL, R03_YJ1022CG, R03_YG1022FC, R03_YJ1022FC, R04_YG1022TC, R04_YJ1022TC, R04_YG1022CL, R04_YJ1022CG, R04_YG1022FC, R04_YJ1022FC, R05_YG1022TC, R05_YJ1022TC, R05_YG1022CL, R05_YJ1022CG, R05_YG1022FC, R05_YJ1022FC  R02R_Y_CUR_GTRAD, R02R_Y_CUR_GRILLO, R02R_Y_GFILTR, R03R_Y_CUR_GTRAD, R03R_Y_CUR_GRILLO, R03R_Y_GFILTR, R04R_Y_CUR_GTRAD, R04R_Y_CUR_GRILLO, R04R_Y_GFILTR, R05R_Y_CUR_GTRAD, R05R_Y_CUR_GRILLO, R05R_Y_GFILTR | Combined aged-up adults to applicable youth variables.  Combined 3 variables for past 30-day use. Quit was defined as reporting past 30 day use in wave N, and no past 30-day use in wave N+1.  Combined exclusive blunting variables (J notation) with cigar variables. |
| Initiation | Adult | R02R_A_NEW_GTRAD, R02R_A_NEW_GRILLO, R02R_A_NEW_GFILTR, R03R_A_NEW_GTRAD, R03R_A_NEW_GRILLO, R03R_A_NEW_GFILTR, R04R_A_NEW_GTRAD, R04R_A_NEW_GRILLO, R04R_A_NEW_GFILTR, R05R_A_NEW_GTRAD, R05R_A_NEW_GRILLO, R05R_A_NEW_GFILTR, | NA |
|  | Youth | R02R_Y_NEW_GTRAD, R02R_Y_NEW_GRILLO, R02R_Y_NEW_GFILTR, R03R_Y_NEW_GTRAD, R03R_Y_NEW_GRILLO, R03R_Y_NEW_GFILTR, R04R_Y_NEW_GTRAD, R04R_Y_NEW_GRILLO, R04R_Y_NEW_GFILTR, R05R_Y_NEW_GTRAD, R05R_Y_NEW_GRILLO, R05R_Y_NEW_GFILTR, | Combined aged-up adults to applicable youth variables. |
| Age | Adult | R01R_A_AGE, R02R_A_AGE, R03R_A_AGE, R04R_A_AGE, R05R_A_AGE | NA |
|  | Youth | R01R_Y_AGE, R02R_Y_AGE, R03R_Y_AGE, R04R_Y_AGE, R05R_Y_AGE | Combined aged-up adults to applicable youth variables. |
| Sex | Adult | R01R_A_SEX, R02R_A_SEX, R03R_A_SEX, R04R_A_SEX, R05R_A_SEX | NA |
|  | Youth | R01R_Y_SEX, R02R_Y_SEX, R03R_Y_SEX, R04R_Y_SEX, R05R_Y_SEX | Combined aged-up adults to applicable youth variables. |
| Race | Adult | R01R_A_RACE, R02R_A_RACE, R03R_A_RACE, R04R_A_RACE, R05R_A_RACE | Recoded into White, Black, Other due to small sample sizes for Asian and Other |
|  | Youth | R01R_Y_RACE, R02R_Y_RACE, R03R_Y_RACE, R04R_Y_RACE, R05R_Y_RACE | Combined aged-up adults to applicable youth variables.  Recoded into White, Other due to small sample sizes for Black, Asian, and Other |
| Ethnicity | Adult | R01R_A_HISP, R02R_A_HISP, R03R_A_HISP, R04R_A_HISP, R05R_A_HISP | NA |
|  | Youth | R01R_Y_HISP, R02R_Y_HISP, R03R_Y_HISP, R04R_Y_HISP, R05R_Y_HISP | Combined aged-up adults to applicable youth variables. |
| State policy | Adult | R01_A_STATE_FIPS, R02_A_STATE_FIPS, R03_A_STATE_FIPS, R04_A_STATE_FIPS, R05_A_STATE_FIPS | Used data from cigar pack quantity and price policy study^1^ to code states based on policy presence for each year |
|  | Youth | R01_Y_STATE_FIPS, R02_Y_STATE_FIPS, R03_Y_STATE_FIPS, R04_Y_STATE_FIPS, R05_Y_STATE_FIPS | Combined aged-up adults to applicable youth variables.  Used data from cigar pack quantity and price policy study^1^ to code states based on policy presence for each year |
| Price | Adult | R01_AG1042TC_D, R01_AG1042CG_D, R01_AG1042FC_D, R02_AG1042TC_D, R02_AG1042TJ_D, R02_AG1042CG_D, R02_AG1042GJ_D, R02_AG1042FC_D, R02_AG1042FJ_D, R03_AG1042TC_D, R03_AJ1042TC_D, R03_AG1042CG_D, R03_AJ1042CG_D, R03_AG1042FC_D, R03_AJ1042FC_D, R04_AG1042TC_D, R04_AJ1042TC_D, R04_AG1042CG_D, R04_AJ1042CG_D, R04_AG1042FC_D, R04_AJ1042FC_D, R05_AG1042TC_D, R05_AJ1042TC_D, R05_AG1042CG_D, R05_AJ1042CG_D, R05_AG1042FC_D, R05_AJ1042FC_D  R01_AG1042TC_C, R01_AG1042CG_C, R01_AG1042FC_C, R02_AG1042TC_C, R02_AG1042TJ_C, R02_AG1042CG_C, R02_AG1042GJ_C, R02_AG1042FC_C, R02_AG1042FJ_C, R03_AG1042TC_C, R03_AJ1042TC_C, R03_AG1042CG_C, R03_AJ1042CG_C, R03_AG1042FC_C, R03_AJ1042FC_C, R04_AG1042TC_C, R04_AJ1042TC_C, R04_AG1042CG_C, R04_AJ1042CG_C, R04_AG1042FC_C, R04_AJ1042FC_C, R05_AG1042TC_C, R05_AJ1042TC_C, R05_AG1042CG_C, R05_AJ1042CG_C, R05_AG1042FC_C, R05_AJ1042FC_C  R01_AG1043TC_D, R01_AG1043CG_D, R01_AG1043FC_D, R02_AG1043TC_D, R02_AG1043TJ_D, R02_AG1043CG_D, R02_AG1043GJ_D, R02_AG1043FC_D, R02_AG1043FJ_D, R03_AG1043TC_D, R03_AJ1043TC_D, R03_AG1043CG_D, R03_AJ1043CG_D, R03_AG1043FC_D, R03_AJ1043FC_D, R04_AG1043TC_D, R04_AJ1043TC_D, R04_AG1043CG_D, R04_AJ1043CG_D, R04_AG1043FC_D, R04_AJ1043FC_D, R05_AG1043TC_D, R05_AJ1043TC_D, R05_AG1043CG_D, R05_AJ1043CG_D, R05_AG1043FC_D, R05_AJ1043FC_D  R01_AG1043TC_C, R01_AG1043CG_C, R01_AG1043FC_C, R02_AG1043TC_C, R02_AG1043TJ_C, R02_AG1043CG_C, R02_AG1043GJ_C, R02_AG1043FC_C, R02_AG1043FJ_C, R03_AG1043TC_C, R03_AJ1043TC_C, R03_AG1043CG_C, R03_AJ1043CG_C, R03_AG1043FC_C, R03_AJ1043FC_C, R04_AG1043TC_C, R04_AJ1043TC_C, R04_AG1043CG_C, R04_AJ1043CG_C, R04_AG1043FC_C, R04_AJ1043FC_C, R05_AG1043TC_C, R05_AJ1043TC_C, R05_AG1043CG_C, R05_AJ1043CG_C, R05_AG1043FC_C, R05_AJ1043FC_C | Combined separate items for price dollars (_D) and cents (_C).  Combined separate items for box price (1042) and single price (1043). Combined exclusive blunting variables (J notation) combined with cigar variables for waves 2-5.  Winsorized: TC: 500 (7 cases); CG: 108.50 (17 cases); FC: 500.23 (5 cases) |
|  | Youth | R02_YG1034TC_D, R02_YG1034TJ_D, R02_YG1034CL_D, R02_YG1034GJ_D, R02_YG1034FC_D, R02_YG1034FJ_D, R03_YG1034TC_D, R03_YJ1034TC_D, R03_YG1034CL_D, R03_YJ1034CG_D, R03_YG1034FC_D, R03_YJ1034FC_D, R04_YG1034TC_D, R04_YJ1034TC_D, R04_YG1034CL_D, R04_YJ1034CG_D, R04_YG1034FC_D, R04_YJ1034FC_D, R05_YG1034TC_D, R05_YJ1034TC_D, R05_YG1034CL_D, R05_YJ1034CG_D, R05_YG1034FC_D, R05_YJ1034FC_D  R02_YG1034TC_C, R02_YG1034TJ_C, R02_YG1034CL_C, R02_YG1034GJ_C, R02_YG1034FC_C, R02_YG1034FJ_C  R03_YG1034TC_C, R03_YJ1034TC_C, R03_YG1034CL_C, R03_YJ1034CG_C, R03_YG1034FC_C, R03_YJ1034FC_C, R04_YG1034TC_C, R04_YJ1034TC_C, R04_YG1034CL_C, R04_YJ1034CG_C, R04_YG1034FC_C, R04_YJ1034FC_C, R05_YG1034TC_C, R05_YJ1034TC_C, R05_YG1034CL_C, R05_YJ1034CG_C, R05_YG1034FC_C, R05_YJ1034FC_C | Combined aged-up adults to applicable youth variables.  Combined separate items for price dollars (_D) and cents (_C).  Combined exclusive blunting variables (J notation) with cigar variables.  Winsorized: CG: 200 (4 cases) |
| Flavors | Adult | R01_AG1050TC, R01_AG1050CG, R01_AG1050FC, R02_AG1130TC, R02_AG1130CG, R02_AG1130FC, R03_AG1130TC, R03_AJ1130TC, R03_AG1130CG, R03_AJ1130CG, R03_AG1130FC, R03_AJ1130FC, R04_AG1130TC, R04_AJ1130TC, R04_AG1130CG, R04_AJ1130CG, R04_AG1130FC, R04_AJ1130FC, R05_AG1130TC, R05_AJ1130TC, R05_AG1130CG, R05_AJ1130CG, R05_AG1130FC, R05_AJ1130FC | Combined exclusive blunting variables (J notation) with cigar variables for waves 3-5. |
|  | Youth | R02_YG1130TC, R02_YG1130CL, R02_YG1130FC, R03_YG1130TC, R03_YJ1130TC, R03_YG1130CL, R03_YJ1130CG, R03_YG1130FC, R03_YJ1130FC, R04_YG1130TC, R04_YJ1130TC, R04_YG1130CL, R04_YJ1130CG, R04_YG1130FC, R04_YJ1130FC, R05_YG1130TC, R05_YJ1130TC, R05_YG1130CL, R05_YJ1130CG, R05_YG1130FC, R05_YJ1130FC | Combined aged-up adults to applicable youth variables.  Combined exclusive blunting variables (J notation) with cigar variables for waves 3-5. |
| Other tobacco use | Adult | R01_AC1003, R01_AP1003, R01_AH1003, R01_AE1003, R01_AS1003SU, R01_AS1003SM, R01_AG1003TC, R01_AG1003CG, R01_AG1003FC, R02_AC1003, R02_AP1003, R02_AH1003, R02_AO1003C, R02_AS1003SM, R02_AG1003TC, R02_AG1003TJ, R02_AG1003CG, R02_AG1003GJ, R02_AG1003FC, R02_AG1003FJ, R03_AC1003, R03_AP1003, R03_AH1003, R03_AV1003EC, R03_AS1003, R03_AG1003TC, R03_AG1003TJ, R03_AG1003CG, R03_AG1003GJ, R03_AG1003FC, R03_AG1003FJ, R04_AC1003, R04_AP1003, R04_AH1003, R04_AV1003, R04_AS1003, R04_AG1003TC, R04_AG1003TJ, R04_AG1003CG, R04_AG1003GJ, R04_AG1003FC, R04_AG1003FJ, R05_AC1003, R05_AP1003, R05_AH1003, R05_AV1003, R05_AS1003, R05_AG1003TC, R05_AG1003TJ, R05_AG1003CG, R05_AG1003GJ, R05_AG1003FC, R05_AG1003FJ | Combined items to create other tobacco product use variables. |
|  | Youth | R02_YC1112, R02_YH1112, R02_YE1112, R02_YS1112SM, R02_YG1112TC, R02_YG1112CL, R02_YG1112FC, R03_YC1112, R03_YH1112, R03_YV1112, R03_YS1112, R03_YG1112TC, R03_YJ1112TC, R03_YG1112CL, R03_YJ1112CG, R03_YG1112FC, R03_YJ1112FC, R04_YC1112, R04_YH1112, R04_YV1112, R04_YS1112, R04_YG1112TC, R04_YJ1112TC, R04_YG1112CL, R04_YJ1112CG, R04_YG1112FC, R04_YJ1112FC, R05_YC1112, R05_YH1112, R05_YV1112, R05_YS1112, R05_YG1112TC, R05_YJ1112TC, R05_YG1112CL, R05_YJ1112CG, R05_YG1112FC, R05_YJ1112FC | Combined aged-up adults to applicable youth variables.  Combined items to create other tobacco product use variables. |
| Brand | Adult | R01_AG1048TC_BRAND, R01_AG1048CG_BRAND, R01_AG1048FC_BRAND, R02_AG1048TC_BRAND, R02_AG1048TJ_BRAND, R02_AG1048CG_BRAND, R02_AG1048GJ_BRAND, R02_AG1048FC_BRAND, R02_AG1048FJ_BRAND, R03_AG1048TC_BRAND, R03_AJ1048TC_BRAND, R03_AG1048CG_BRAND, R03_AJ1048CG_BRAND, R03_AG1048FC_BRAND, R03_AJ1048FC_BRAND, R04_AG1048TC_BRAND, R04_AJ1048TC_BRAND, R04_AG1048CG_BRAND, R04_AJ1048CG_BRAND, R04_AG1048FC_BRAND, R04_AJ1048FC_BRAND, R05_AG1048TC_BRAND, R03_AJ1048TC_BRAND, R05_AG1048CG_BRAND, R05_AJ1048CG_BRAND, R05_AG1048FC_BRAND, R05_AJ1048FC_BRAND | Combined exclusive blunting variables (J notation) with cigar variables for waves 2-5.  Used to differentiate between premium and large cigars.  Premium brands listed in PATH: Arturo Fuento, Ashton, CAO, Cohiba, Davidoff, Don Tomas, La Corona, Macanudo, Makers Mark, Nat Sherman, Padrom, Partagas, Perdomo, Punch, Rocky Patels, Romeo y Julieta. For those with “other” selected, price was used to differentiate. |
|  | Youth | R02_YG1048TC_BRAND, R02_YG1048TJ_BRAND, R02_YG1048CL_BRAND, R02_YG1048GJ_BRAND, R02_YG1048FC_BRAND, R02_YG1048FJ_BRAND, R03_YG1048TC_BRAND, R03_YJ1048TC_BRAND, R03_YG1048CL_BRAND, R03_YJ1048CG_BRAND, R03_YG1048FC_BRAND, R03_YJ1048FC_BRAND, R04_YG1048TC_BRAND, R04_YJ1048TC_BRAND, R04_YG1048CL_BRAND, R04_YJ1048CG_BRAND, R04_YG1048FC_BRAND, R04_YJ1048FC_BRAND, R05_YG1048TC_BRAND, R03_YJ1048TC_BRAND, R05_YG1048CL_BRAND, R05_YJ1048CG_BRAND, R05_YG1048FC_BRAND, R05_YJ1048FC_BRAND | Combined aged-up adults to applicable youth variables.  Combined exclusive blunting variables (J notation) with cigar variables.  Used to differentiate between premium and large cigars. |
| Notes: Analytic code available upon reasonable request to corresponding author. Youth data include aged-up adult data; variables merged include those applicable from adult list. Item wording and response options can be obtained from the Restricted Use File Codebooks available for download at https://www.icpsr.umich.edu/web/NAHDAP/studies/36231.  ^1^Jensen JL, Delnevo CD, Merten JW, Torton B, Azagba S. A synthesis of local cigar pack policies in the US. Preventive medicine reports. 2022 Aug 1;28:101865. | | | |
